# Optimal Anticoagulation for Massive Left Atrial Appendage Thrombus in Atrial Fibrillation With a History of Gastrointestinal Bleeding

**DOI:** 10.7759/cureus.94430

**Published:** 2025-10-12

**Authors:** Van Thi Ai Hoang, Huynh Thuy Tien Dinh, Vinh Sieu Lam, Phuong Nguyen Thao Le, Phillip Tran

**Affiliations:** 1 International Medicine, Nam Can Tho University, Can Tho, VNM; 2 Cardiovascular Research, Methodist Hospitals, Merrillville, USA; 3 Faculty of Medicine, Moscow State University of Medicine and Dentistry Named After A.I. Yevdokimov, Moscow, RUS; 4 Medicine, The University of Texas at Austin, Austin, USA; 5 Cardiovascular, Yavapai Regional Medical Center, Prescott, USA

**Keywords:** anticoagulation strategy, atrial fibrillation, left atrial appendage thrombus, thrombus mobility, warfarin therapy

## Abstract

Systemic embolism is a serious complication of atrial fibrillation (AF), most commonly originating from thrombi in the left atrial appendage (LAA). Thrombi that are large (≥15 mm) or exhibit mobility carry particularly high embolic risk, yet management can be challenging in patients with elevated bleeding risk. Advanced echocardiographic techniques now allow quantification of thrombus mobility, and dense spontaneous echo contrast (SEC) on transesophageal echocardiography has been identified as an independent predictor of thromboembolic events. We report the case of an 80-year-old woman with paroxysmal AF, non-Hodgkin lymphoma in remission, hypothyroidism, and a history of perforated gastric ulcer, in whom a large, mobile LAA thrombus (8 × 15 mm) was incidentally detected and confirmed by transesophageal echocardiography. Anticoagulation with warfarin, titrated to an INR of 2.5-3.0, led to complete thrombus resolution within six weeks. This case illustrates the substantial embolic risk associated with mobile LAA thrombi and demonstrates that individualized anticoagulation with careful monitoring can achieve safe and effective thrombus resolution in patients at high risk of bleeding. The novelty lies in highlighting individualized management as a viable strategy in this complex clinical setting, providing an important teaching point for practice.

## Introduction

Atrial fibrillation (AF) is the most common sustained arrhythmia, affecting over 37 million people worldwide and increasing in prevalence with age and comorbidities such as hypertension and structural heart disease [1]. AF increases the risk of ischemic stroke fivefold [1], with the left atrial appendage (LAA) being the source of ~90% of thrombi in nonvalvular AF [2,3]. The trabeculated anatomy of the LAA promotes blood stasis and thrombus formation, particularly when atrial contractility is impaired [4].

LAA thrombus (LAAT) is a major cause of cardioembolic events and is often detected incidentally on transesophageal echocardiography (TEE) performed before cardioversion or ablation [5,6]. Anticoagulation remains the cornerstone of treatment. Vitamin K antagonists such as warfarin have long been standard therapy [7], while direct oral anticoagulants (DOACs) are increasingly used and have shown comparable efficacy in thrombus resolution [8].

We present the case of an 80-year-old woman with paroxysmal AF and a history of perforated gastric ulcer, found to have a large, mobile LAAT that resolved completely after six weeks of warfarin therapy. This case is unique because the complete resolution of a large, mobile thrombus in a patient with significant bleeding risk is uncommon. Warfarin was preferred over DOACs in this context because INR monitoring allowed careful titration of anticoagulation intensity, providing greater control in a patient with prior gastrointestinal bleeding risk. The case supports clinical practice by highlighting the importance of individualized anticoagulation strategies in complex, high-risk patients.

## Case presentation

An 80-year-old woman, with a medical history significant for paroxysmal AF, non-Hodgkin lymphoma in remission, hypothyroidism, and a history of perforated gastric ulcer, was admitted for further evaluation after a LAAT was detected incidentally. The thrombus was first identified on a routine follow-up computed tomography (CT) scan performed for surveillance purposes.

**Figure 1:**
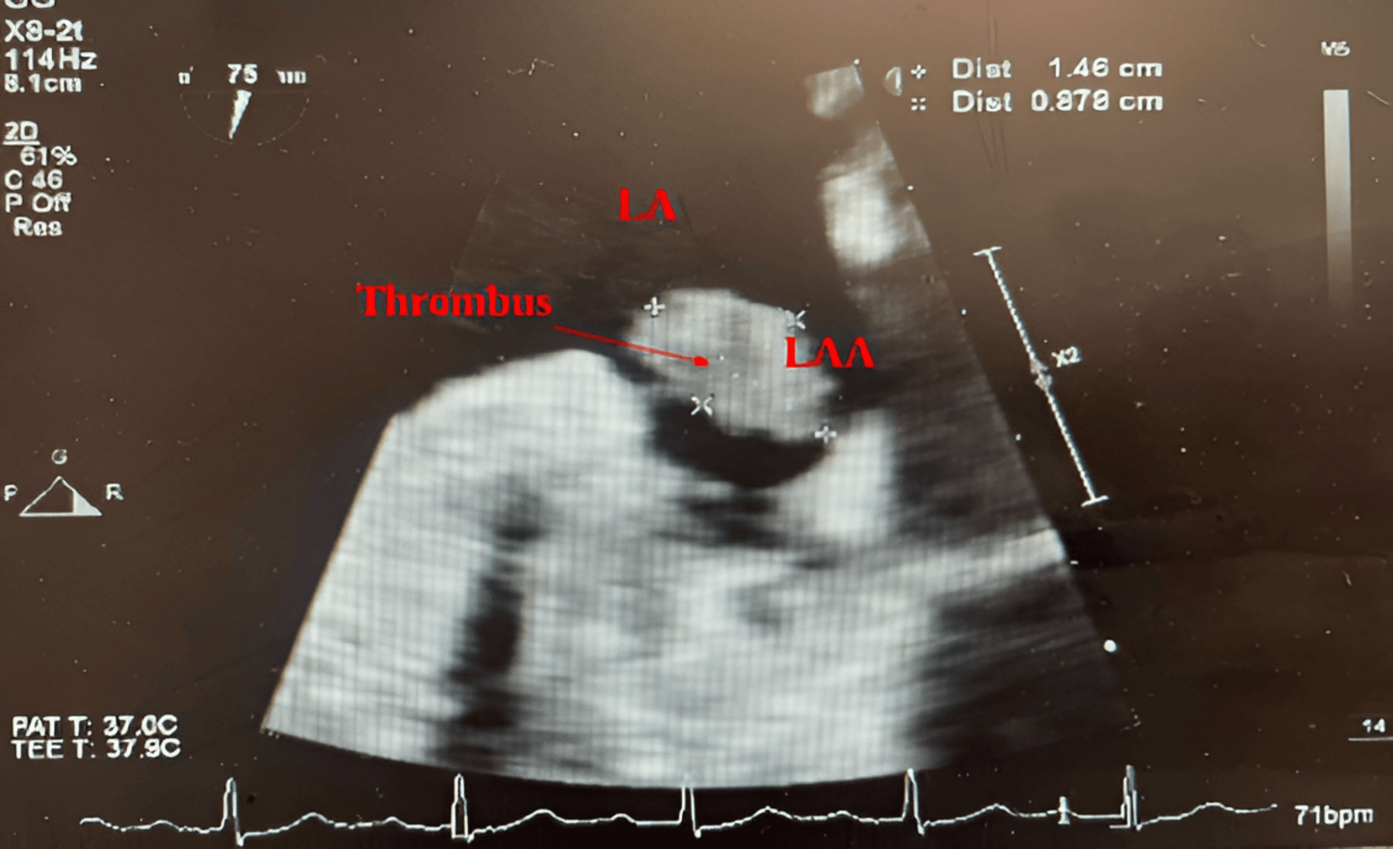
Transesophageal echocardiogram in 2D mode showing a large, mobile thrombus in the left atrial appendage, measuring 8 x 15 mm. The thrombus appears as a hyperechoic mass with defined borders.

**Figure 2:**
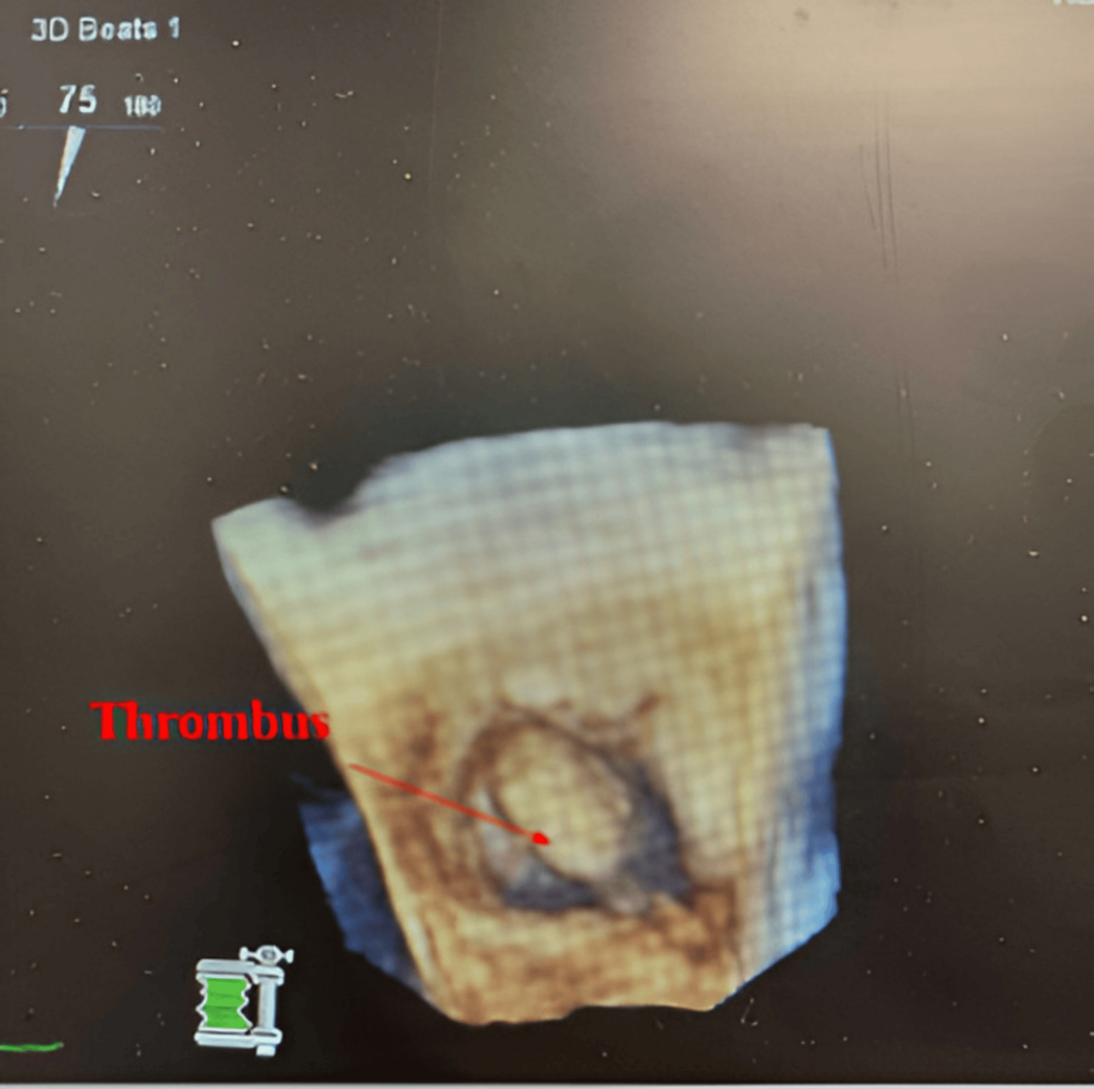
Transesophageal echocardiogram - zoomed view in 3D mode demonstrating the left atrial appendage thrombus measuring 8 x 15 mm with clear visualization of its attachment point and mobility.

To further characterize the finding, a TEE was performed. This confirmed the presence of a large, mobile thrombus within the left atrial appendage, measuring 8 × 15 mm (Figures 1-2). Given the thrombus size and mobility, anticoagulation therapy was indicated to reduce the risk of systemic embolization.

Considering the patient’s underlying conditions, particularly her prior history of perforated gastric ulcer, warfarin was selected as the anticoagulant of choice. The target international normalized ratio (INR) was set at 2.5-3.0 to optimize thrombus resolution while balancing bleeding risk. The patient was managed as an inpatient during the initiation phase, and her INR was closely monitored during therapy.

**Figure 3:**
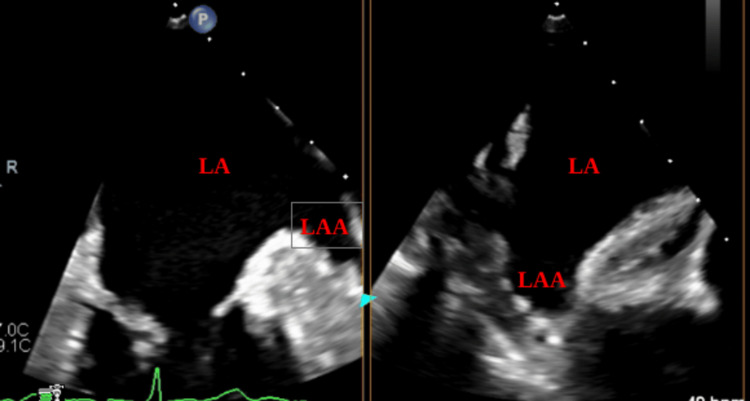
Transesophageal echocardiogram after six-week anticoagulation treatment showing complete resolution of the left atrial appendage thrombus with clear left atrial appendag (LAA) cavity.

After six weeks of continuous warfarin treatment, a repeat TEE was performed. The follow-up imaging demonstrated complete resolution of the thrombus (Figure 3), and no thromboembolic events were recorded during the treatment period.

## Discussion

The incidental finding of a large, mobile thrombus in the LAA in this patient with paroxysmal AF represented a substantial risk for systemic embolism. Thrombus size and mobility are recognized predictors of embolic events, with mobile thrombi particularly prone to detachment and causing ischemic stroke or peripheral embolization [5,6]. Prompt initiation of effective anticoagulation is therefore critical in such cases.

The choice of anticoagulant was guided by both thrombus morphology and the patient’s comorbidities. Low-molecular-weight heparin (LMWH) has limited evidence for long-term left atrial appendage thrombus resolution compared with warfarin or DOACs, and its use is further constrained by the need for renal dose adjustment, making it less suitable for chronic therapy. DOACs are increasingly employed for LAA thrombus due to their predictable pharmacokinetics, favorable safety profile, and lack of routine monitoring requirements [9-11]. However, in this patient, warfarin was chosen because of a history of a perforated gastric ulcer, which significantly increased the risk of gastrointestinal bleeding [12]. Evidence indicates that certain DOACs - particularly dabigatran and rivaroxaban - may be associated with a higher incidence of GI bleeding compared with warfarin in elderly populations [13]. Warfarin offers individualized INR monitoring, dose adjustment, and rapid reversal with vitamin K in case of hemorrhage, providing a safety margin in high-bleeding-risk patients [14].

INR target selection was a key part of management. While the standard therapeutic INR range for AF is 2.0-3.0 [10], higher targets have been suggested for large or mobile thrombi to increase anticoagulant efficacy [8]. In this case, an INR of 2.5-3.0 was chosen as a balance between maximizing thrombus resolution and minimizing bleeding risk. Avoiding full escalation to 3.0-3.5 was deemed prudent given the patient’s gastrointestinal bleeding risk.

Treatment outcome was favorable - complete thrombus resolution was achieved after six weeks of warfarin therapy without bleeding events. This aligns with prior studies reporting thrombus resolution rates of 60-80% with warfarin over 4-12 weeks [7,14]. While several observational studies and meta-analyses suggest that DOACs achieve similar or faster thrombus resolution [9,10], robust randomized data in this specific setting are still limited. Complete resolution of such a large and mobile thrombus in the LAA within six weeks is uncommon, particularly in a patient with a significant gastrointestinal bleeding risk. This underscores the rarity of the case and provides an instructive example of how individualized anticoagulation can achieve successful outcomes in high-risk scenarios.

Interventional options, such as percutaneous LAA occlusion devices (e.g., Watchman), have been shown to reduce stroke risk in AF [15,16], but are contraindicated in the presence of active thrombus due to the risk of procedural embolization [3,15,16]. In this patient, anatomical and clinical factors also precluded such interventions, further supporting optimized medical management.

This case highlights the importance of prompt recognition and tailored anticoagulation in managing large, mobile LAATs. Repeat imaging remains essential to confirm thrombus resolution before any rhythm-control interventions are considered.

## Conclusions

This case highlights that warfarin, with an individualized INR target, can be a safe and effective therapy for resolving large, mobile LAA thrombi in patients with elevated bleeding risk. Although DOACs are increasingly favored, warfarin remains a valuable option in scenarios requiring close monitoring or rapid reversal. Ultimately, individualized anticoagulation strategies and vigilant follow-up are essential to achieving optimal outcomes.
